# Knowledge and practices that constitute nurses in Primary Care

**DOI:** 10.1590/0034-7167-2025-0083

**Published:** 2026-07-31

**Authors:** José Ricardo Guimarães dos Santos, Stefanie Griebeler Oliveira, Teila Ceolin, Fernanda Eisenhardt de Mello, Sidneia Tessmer Casarin, Franciele Roberta Cordeiro, Vania Dias Cruz, Adrize Rutz Porto

**Affiliations:** I Prefeitura de Alvorada. Alvorada, Rio Grande do Sul, Brazil; II Universidade Federal de Pelotas. Pelotas, Rio Grande do Sul, Brazil; III Universidade Federal do Pampa. Uruguaiana, Rio Grande do Sul, Brazil

**Keywords:** Nurses, Primary Health Care, Primary Care Nursing, Nursing, Practical, Health Policy., Enfermeras y Enfermeros, Atención Primaria de Salud, Enfermería de Atención Primaria, Enfermería Práctica, Política de Salud.

## Abstract

**Objectives:**

to analyze the knowledge and practices that constitute primary care nurses in a municipality in southern Brazil.

**Methods:**

a qualitative, Foucauldian-inspired study was conducted with 31 nurses from Basic Health Units between January and June 2022. Data were managed using ATLAS.ti^®^ software and subjected to content analysis and problematization using Foucault’s tools such as knowledge, power, discourse, and subjectivation.

**Results:**

nurses’ knowledge and practices are shaped by public policies, especially those directed at the bodies of women and children. They are included in the organization and functioning of programmatic actions, guiding them towards a network-oriented approach.

**Final Considerations:**

nurses act as biopolitical agents, guiding state actions towards managing the population’s lives. The overload is a result of the pressure to meet health indicators, multifunctionality, and healthcare computerization. Decentralizing responsibilities common to other team members can minimize this overload.

## RESUMEN

**Objetivos:**

analizar los conocimientos y prácticas que constituyen a las enfermeras de atención primaria en un municipio del sur de Brasil.

**Métodos:**

se realizó un estudio cualitativo de inspiración foucaultiana con 31 enfermeras de Unidades Básicas de Salud entre enero y junio de 2022. Los datos se gestionaron mediante el software ATLAS.ti^®^ y se sometieron a análisis de contenido y problematización utilizando herramientas foucaultianas como conocimiento, poder, discurso y subjetivación.

**Resultados:**

los conocimientos y las prácticas de las enfermeras se ven influenciados por las políticas públicas, especialmente las dirigidas a las mujeres y los niños. Se les incluye en la organización y el funcionamiento de las acciones programáticas, orientándolas hacia un enfoque en red.

**Consideraciones Finales:**

las enfermeras actúan como agentes biopolíticos, guiando las acciones del Estado hacia la gestión de la vida de la población. La sobrecarga se debe a la presión para cumplir con los indicadores de salud, la multifuncionalidad y la informatización de la atención médica. Descentralizar las responsabilidades comunes a otros miembros del equipo puede minimizar esta sobrecarga.

## INTRODUCTION

Throughout history, nursing has had different configurations, acquiring a scientific character internationally in modern times, in the 19^th^ century with Florence Nightingale, and in Brazil with the creation of *Escola de Enfermeiras do Departamento Nacional de Saúde Pública* in 1922, currently known as *Escola Anna Nery* ^(1)^. This path is marked by a biological, hospital-centric, and reductionist view of knowledge, organized and disciplined, spanning periods of war and changes in the political landscape until the Brazilian Sanitary Reform, which (re)formulated Brazilian policies, modifying the role of nurses^(2)^.

Another milestone in the history of Brazilian nursing was the first edition of the Brazilian National Primary Care Policy (In Portuguese, *Política Nacional de Atenção Básica* - PNAB), which presented the general principles of Primary Care (PC) within the Brazilian Health System (In Portuguese, *Sistema Único de Saúde* - SUS), recognizing nurses as indispensable in Family Health teams. Their assigned responsibilities ranged from individual, collective, and managerial care to participation in social control^(3)^.

Currently, after two editions of PNAB, nurses’ legitimacy in performing educational, care, and administrative activities is recognized, since they are responsible for conducting home visits, nursing consultations, territory diagnosis, welcoming with qualified listening and risk classification, according to established protocols, as well as planning, managing, and assessing the actions developed by nursing technicians/assistants and community health workers (CHWs)^(4)^.

Supported by public policies, nurses begin to intervene in biological domains, backed by scientific and technical knowledge that establishes itself as a form of knowledge and power, creating mechanisms of control and domination over individuals, from healthcare services to the home^(5)^. Thus, studying the knowledge and practices that constitute PC nurses allows for the observation of policies that are more or less consolidated, with greater engagement and involvement of professionals in their guidelines and actions. It also allows for an examination of the organization, functioning, and shifting of programs, policies, or networks.

Considering that nurses practice their profession following SUS guidelines, it is interesting to listen to and analyze these trajectories. This problematization tends to denaturalize and identify the shifts that policies and programs undergo over time. It is believed that policies that have affected certain bodies for a longer period tend to be more crystallized in the practices and knowledge that constitute professionals. Therefore, the intention of this study is not to construct a history that shows a linearity in PC nurses’ performance, but to point out the ruptures, shifts, and movements in the formation of certain knowledge and practices exercised by them in this space. Given this, the research question was defined as: what knowledge and practices constitute the PC nurses of a municipality in southern Brazil?

## OBJECTIVES

To analyze the knowledge and practices that constitute PC nurses in a municipality in southern Brazil.

## METHODS

### Ethical aspects

The data presented are part of a larger research project, which deals with the history of nurses in a municipality in southern Brazil, and was approved by a Research Ethics Committee, under Opinion 5,173,063. The ethical precepts of Resolution 466/2012^(7)^ were respected. Participants were identified by code, with the letter N (for nurse), followed by the numerical order of the interviews.

### Study design

This is qualitative research from a problematizing perspective inspired by Foucault. Problematization is characterized by the elaboration of a domain of facts, practices, and thoughts, which critically suspend political actions and consider the historical period^(6)^. COnsolidated criteria for REporting Qualitative research criteria were followed.

### Study setting

The research setting consisted of the Basic Health Units (BHUs) of a municipality in southern Brazil, and the data collection period was between January and June 2022. There are 51 BHUs, 38 in the urban area and 13 in the rural area. Among these, 38 have a Family Health Strategy (FHS), and 11 are traditional models, one with a mixed FHS model - a BHU that also provides night-time care to the population - and one with a Community Health Worker Strategy (CHWS). This research included 49 of these BHUs, as it did not include the mixed FHS and CHWS models.

### Data source

The macro-project aimed to involve one nurse from each BHU, whether conventional or FHS. Inclusion criteria comprised having at least one year of work experience in FHS or conventional BHU and being a nurse with the longest experience in PC. In BHUs with more than one nurse meeting the criteria, the nurse with the longest experience in PC in the municipality was considered. The exclusion criterion included being on leave or vacation.

### Data collection and organization

An invitation to participate in the research was made by telephone call, messages via the WhatsApp^®^ application, or in-person contact at BHUs. Towards the end of the data collection period, BHUs that had not responded to previous contacts received an additional 21 contact attempts, with 13 contacts made via instant messaging using WhatsApp^®^, four by cell phone call, and four in-person contacts. Of the units contacted, only five agreed to participate and six did not respond, which was considered a refusal. Therefore, 31 nurses participated, while 18 did not express interest in participating.

Fieldwork was conducted by eight nursing students, who received four hours of training to participate in this stage. Semi-structured interviews were used as the data collection technique, conducted either in person with a voice recorder, or online via the Google Meet^®^ platform, using a mobile phone as a voice recorder. The interview was composed of four axes: axis 1 - participant characterization; axis 2 - contextualization of public health policies; axis 3 - work practice; and axis 4 - work environment. The mean time between interviews was 32 minutes, and for greater reliability of the statements, they were transcribed in full using Microsoft Word^®^, totaling approximately 13 hours of interviews and 202 pages of content. To compile the empirical material used in this research, information relating to axes one through four was extracted from the interviews conducted.

### Data analysis

All the information produced was coded using ATLAS.ti^®^ cloud version. The codes were themes that emerged from content analysis stages^(8)^, developed around three chronological poles: pre-analysis - a preliminary reading based on initial contact with excerpts from the interviews, drawing on first impressions related to the objectives, which aided in coding using software; material exploration - reading, deepening, and organizing the analysis, confirming the identified categories and subcategories; data processing and interpretation - transforming raw data into meaningful data, allowing the construction of structures that condense and highlight this information. Four codes were generated, namely historicity, practice, ruptures, and knowledge, which were problematized using Foucault’s theories of knowledge, power, discourse, and subjectivation. After these stages, categories were constructed.

## RESULTS

In this study, 31 participants were interviewed, and their characteristics are presented in Chart 1.

**Chart 1:** Sociodemographic characteristics of study nurses, Pelotas, Rio Grande do Sul, Brazil, 2022

Code	Length of training (years)	Age	Biological sex	Self-declared color	Health unit model	Workload
N1	14	40	Woman	White	FHS	40 hours
N2	8	33	Woman	Black	Rural traditional	30 hours
N3	7	36	Woman	White	FHS	40 hours
N4	15	39	Woman	White	FHS	30 hours
N5	8	30	Woman	Black	FHS	40 hours
N6	15	47	Woman	White	FHS	40 hours
N7	9	31	Woman	White	FHS	40 hours
N8	22	45	Woman	White	Traditional	30 hours
N9	15	35	Woman	White	FHS	40 hours
N10	8	30	Woman	White	Rural FHS	40 hours
N11	15	38	Woman	White	FHS	40 hours
N12	20	42	Woman	White	Traditional	40 hours
N13	10	52	Woman	White	Rural FHS	40 hours
N14	22	51	Woman	White	FHS	40 hours
N15	6	30	Woman	White	FHS	40 hours
N16	12	38	Woman	White	FHS	40 hours
N17	16	40	Woman	Brown	FHS	40 hours
N18	26	50	Woman	White	Traditional	36 horas
N19	18	44	Woman	White	FHS	40 hours
N20	20	41	Woman	White	FHS	40 hours
N21	4	28	Woman	White	FHS	40 hours
N22	15	38	Woman	White	FHS	40 hours
N23	21	43	Woman	White	FHS	40 hours
N24	12	48	Man	White	Rural FHS	40 hours
N25	8	34	Woman	White	FHS	40 hours
N26	8	37	Woman	White	FHS	40 hours
N27	28	56	Woman	White	FHS	40 hours
N28	11	47	Woman	Brown	Traditional	30 hours
N29	36	57	Woman	White	Traditional	40 hours
N30	12	39	Woman	White	FHS	30 hours
N31	35	57	Woman	White	Rural FHS	40 hours

*FHS - Family Health Strategy; N - nurse.*

Among participants, 30 (96.7%) are women, aged between 28 and 57 years, with the majority (87%) self-identifying as white. Furthermore, 87% of nurses have a statutory employment contract. Concerning working hours, the majority (80.6%) work 40 hours per week. Concerning the type of BHU where they work, 80.6% work in FHS units, and 19.3% in traditional BHU units, with 12.9% working in rural FHS units and 3.2% in traditional rural units.

The following categories will be presented below: Historicity: Primary Care policies - “from drawers to networks”; Records, computerization and bureaucratization - “the e-SUS works when it wants to”; Conventional “open door” Basic Health Unit and the (im)possibilities in the Family Health Strategy; and Multifunctionality of nurses in the Family Health Strategy versus the “minimum infrastructure”.

### Historicity: Primary Care policies - “from drawers to networks”

When reflecting on the (de)construction of existing health policies during their length of service and their impacts throughout their careers, nurses shared their perceptions regarding the changes in their responsibilities and practices. Initially, with actions or programs aimed at a specific population, the formulation of policies shows that they have the effect of directing and guiding nurses to organize and structure the provision of care for certain populations.

*Right from the start, until quite recently, it seemed like little boxes: child health, women’s health. Men’s health was hardly ever mentioned, it wasn’t discussed at all, it was treated like adult health. Sexually transmitted diseases, or AIDS, are now STIs.* (N12)

*Initially, this was the program that existed: childcare, which was the care of young children, with vaccination, monitoring of growth and development, and prenatal care for pregnant women. And programs were added over time, later with the municipalization of the programs.* (N29)

*It improved a lot when the Stork Network started, it helped us a lot, you know, to structure the women’s and children’s healthcare service. My training was very different, when I did my undergraduate studies, it was totally different, and then with the Stork Network we started to kind of think together, you know, mother and child, and look at things differently.* (N23)

The shift to a network model also expanded nurses’ responsibilities, allowing them to conduct nursing consultations and home visits in the field of care.

*Nurses working in healthcare today, we see nurses doing nursing consultations and home visits, and we think it’s easy, but for us, this was an achievement. Because when we joined the network, we were given five units to be responsible for, to spend one day in each unit.* (N29)

*Then this government, from the PDT, expanded it to 52 units. It was very interesting, but that format was all BHUs, there was no FHP. So, the general practitioner, the pediatrician, sometimes a gynecologist worked there, it was the very basics. There was a vaccination room that was used for everything together for childcare. And for nursing, I think there was another one for procedures.* (N23)

### Records, computerization and bureaucratization - “the e-SUS works when it wants to”

Workplace computerization has led to an overload in professional work. Reports show that there seem to be problems with data storage and, often, the systems do not function well, generating the need for duplicate records in both physical and electronic formats:

*Child health, for instance, we have to know how to organize, see if we have everything up to date, vaccinations. We can’t rely on e-SUS, because it only works when it wants to. I don’t use physical* [medical records], *because I think they should be abolished.* (N14)

*With the e-SUS system, you register and register, and out of 100, only about 40 or 50 are counted.* (N26)

*You lose some of the excellence of the work, because you’re only worried about filling out forms and you don’t look at the child, the mother, the woman who came for PC.* (N14).

Computerization is perceived as a means of control because, through the data recorded in electronic medical records, managers extract information to hold nurses and their respective teams accountable, who perceive these indicators as impossible given their realities.

*What is the feeling I have? That things are coming to an end. That there is a gigantic demand for data control, for achieving goals, for proving services, and then we see a kind of demand and no. If there is support, let’s suppose, I’ve had a micro-area completely without CHWs for five years - the most vulnerable micro-area in the territory. Then I redistributed the CHWs. Last year, one of the best CHWs retired; I was left with two uncovered areas.* (N26)

*This is already official, there will be 12 or 13, but it is not known what the other indicators will be. There are indicators that they have already created so that people don’t achieve the goals. The goal is as follows: you achieved the goal, the municipality will earn X. Didn’t achieve it? You don’t earn.* (N24)

### Conventional “open door” Basic Health Unit and the (im)possibilities in the Family Health Strategy

Some participants work in BHUs in the municipality using the traditional model and, based on their experiences, describe their practices in comparison to those of a FHS unit, as well as some of its limitations, such as not knowing the user profile for planning care provision, not conducting home visits, not having group composition, and not having a complete team due to the absence of CHWs in the staff, which centralizes functions in nurses.

*In the strategy, we have teams that seem more complete* [...] *so you can coordinate better. The new reception protocol* [...] *it was designed so that, for instance, the first care is not done only by nursing staff. It can be carried out by any higher-level professional in the unit* [...] *and we can’t do that; it always ends up being centered on the nurse for many reasons* [...]. (N2)

*I think it’s more interesting, you create bonds. We create bonds, we see the child grow, we see the development, we see some things that we observed and passed on to the doctor and it was resolved because we saw them.* (N8)

*What is the difference? The population itself is not delimited. For instance, in the strategy, you have so many inhabitants. You know how many pregnant women you have, how many children you have, how to organize it in a way that is more practical. Here, for instance, if you ask me how many pregnant women you have, we have an average of 200 pregnant women, but on average.* (N18)

Professionals discussed the reorganization of their routines at the conventional BHU, starting from the premise that they are an “open door” service and need to absorb the demands that arrive, facing challenges in actively seeking out patients and conducting home visits.

*We don’t do home visits. In a traditional unit, there wouldn’t be that need, but when there’s a situation that requires it, we do it because we have a driver.* (N2)

*In the conventional BHU where I am now, we can’t do an active search, even though I have a connection. And since we are “open door”, we receive people from all over, and when it comes to active search, there’s not much we can do. The home visit part is also an activity that is rarely done.* (N12)

When reflecting on the difficulties in their practices within FHS, nurses pointed to the impact of the lack of professionals from different categories as a general (im)possibility for group actions:

*We try to carry out the priority areas of the strategy as much as possible. We miss the groups. And we can’t because of the outdated team.* (N1)

*Right now, we are overloaded and also have a shortage of colleagues, of all professionals. There is no complete team in the city.* (N23)

*I, luckily, currently have a doctor, but to give you an idea, several teams in the rural area, with incomplete teams, don’t have a doctor, that is, it becomes difficult to work, to carry out a Family Health Program if the team is incomplete.* (N24)

The changes foreseen in public policies, observed throughout the years of practice, were also pointed out by nurses as creating obstacles in their work. Beyond the difficulties in implementation, these policies often follow paths contrary to those proposed in their creation, causing insecurity by preventing them from acting according to guidelines and manuals.

*My impression is that SUS is ending, that FHS is ending. That’s the feeling I have every day when I come to work: that we’re trying to hold onto a sinking boat. A leaky boat, and we’re trying to bail out the water with buckets, but more water gets in than we can bail out.* (N23)

*I think health policies, especially family health policies, are very important, but in recent years they have been undergoing major changes, including the Family Health Strategy, which I see seems to be losing its direction. Things are changing a lot, and I’m worried about the future of family health.* (N24)

However, some statements demonstrate that, despite the challenges in FHS, there are possibilities and potential found in its practices to carry out certain aspects, even if different from what should be and/or is written in PC policy guidelines. As examples, they cited groups, the Health at Schools Program, and home visits.

*Here, we put all public policies into practice, not in the way it should be, but yes, we have childcare, where we implement the Child Health Booklet, the Basic Care Booklet. So, we try to carry out everything that is included there. In women’s health, the same thing. In cytopathological collections, in low-risk prenatal care, we do all this follow-up and use the care booklets, which is the material we have from the ministry.* (N5)

*Now the group of pregnant women is starting again. We can’t do much. There is the HSP, where we do health in schools. We have six schools linked to our unit, so we give more attention to these children as well. Home visits, we do them every Thursday afternoon. If there is a patient or any demand that comes from the street, we organize ourselves and leave at 11:00 to organize the visits.* (N7)

Furthermore, nurses recognize FHS as a strategy that promotes a stronger bond with the user, requiring greater responsibility, knowledge of their family, the context, health situation, and CHWs - the individuals who mediate this relationship.

*In FHS, we are much more connected with the family. The responsibility is much greater* [...] *CHWs bring us the demands* [...]. (N4)

*The presence of CHWs when they are active makes a big difference in creating a bond. They bring up many issues in meetings, for instance, when a person comes for an appointment and you meet the person at that moment, but you don’t know their context. So, a CHW says, “Look, there’s no way someone can prescribe such and such medication for such and such patient, because their house is this small and they barely have anything to eat, and the patient is ashamed to say so”. Some people say, “Look, if this medicine is expensive, I can’t afford to buy it”, but some people don’t say that.* (N5)

### Multifunctionality of nurses in the Family Health Strategy versus the “minimum infrastructure”

The many roles played by PC nurses are also brought into play in their statements, highlighting the centralization of administrative responsibilities in these professionals, who are responsible for managing BHU, its supplies, and the maintenance of the facility alongside their clinical practice.

*Because everything falls on the nurse’s shoulders. They don’t distribute the tasks. Everything new that comes up is the nurse’s responsibility, from requesting a lightbulb replacement to a low-risk prenatal care appointment, and they want us to handle it all in those eight hours of work per day, and then, sometimes, it becomes very difficult. You lose a whole morning with patient intake, and then there’s the time when you have to process requests, when you have to manage the Pap smears* [cervical cytology exams]. *There are several administrative demands that are extremely overwhelming.* (N3)

*I have a ton of reports to do, schedules to make. I have to do the timekeeping; I have to* [...] *an employee went on vacation, on leave for who knows what, we have to deal with the bureaucracy. There’s not much time left for us* [...] *we end up dealing more with the demand, putting out fires than what comes in. Everything goes through the nurse: it’s the child who arrives with a fever, who has bronchitis, it’s a little wound, it’s everything.* (N9)

*The thing is, nurses, in the daily routine of a unit, manage everything. From medication orders, supply orders, repairs, maintenance, controls, who the pregnant women are. Whether they are coming or not. Who the children in well-child care are. Having that support, knowing that CHWs are handling their area and are able to fill in for those in other areas that don’t have anyone.* (N26)

In addition to the many functions performed, the relationship with the physical space and the impossibility of offering care foreseen by current policies and protocols, such as welcoming and organizing the space to allow air circulation and optimize service to all users arriving at the location, are highlighted in the following statements:

[...] *I believe that, given the number of professionals working here, we could have a larger unit. We have two nursing rooms: one for vaccinations and another for nursing consultations. There are four nurses per shift, so we don’t have enough space for everyone to work. We even end up sitting in the pharmacy* [...] *we need an expansion to accommodate the team. For users, it would reduce waiting times* [...]. (N4)

[...] *we have minimal infrastructure. We have professionals to provide care; we lack physical space; we cannot attend to people according to the necessary protocol, for instance, attending to people in a room with the door closed. Ventilation: there are rooms that we adapted that have no windows or doors, so we can at least listen to a patient and optimize our time* [...]. (N11)

[...] *there’s no nursing room* [...] *right now, they want to implement the triage process, but they don’t have a room for triage. Nurses don’t have their own rooms, and this is something that has been trivialized for years* [...]. (N14)

## DISCUSSION

There is a predominance of women among participants, which tends to lead them to cite programmatic actions for women and children. The mean length of training was 12 years, which allows us to identify changes in the organization and functioning of programmatic actions with a focus on network orientation. This broadens nurses’ perspective on the people assigned to their area of practice. Among the challenges, professionals’ overload due to computerization and bureaucratization, and nurses’ multifunctionality stand out.

Concerning the organizational changes in the healthcare service, the mention of “little drawers”, which included men, women, and children, paints a picture of fragmented healthcare. From specific programs to healthcare networks, it is noted that discursive and non-discursive practices intertwine to increase the population’s health levels. In this regard, PC nurses, working in family health or conventional healthcare, demonstrate that the most frequently performed actions included childcare and cervical cancer prevention^(9)^.

The Stork Network was cited by nurses as an example of a policy that allowed for the structuring of care and surveillance over the woman’s body, emerging based on axes^(10)^ of humanization and comprehensiveness in health. In its ten years of implementation^(11)^, it is considered effective, as it included the presence of a partner or other family member chosen by women, consisting of a structure^(12)^ composed of places and agents aimed at attending to low-risk pregnancy, childbirth, and postpartum. For Foucault^(13)^, the first bodies that received strategic interventions aimed at their productivity were healthy women and the births of babies with the same vitality.

Biopolitics targets the human species and the population, and is a technology of power that aims to regulate daily life. This form of power is used by various institutions, operating through segregation, the organization of bodies, and social hierarchization, guaranteeing relations of domination^(14)^. In this regard, the organization of health policies into networks enables the exercise of biopolitics among healthcare professionals, who manage the population’s health levels, even with persistent traces of a fragmented system.

Nurses discussed health models and their transitions, exemplifying how the Family Health Program was structured with defined objectives, later becoming a strategy that encompassed new ways of (re)producing health, understanding the territory, and the profile of individuals. However, the set of demands placed on nurses is not fully met, since they are responsible for integrating into the health team and also developing their professional practice^(9)^.

Configurations in these policies have a direction, which is the population. For Foucault^(15)^, the population does not consist of the individual-body, but rather a body with countless heads, if not infinite, at least necessarily countable. To know these bodies, there is the exercise of power, which permeates, produces things, forms knowledge and produces discourse. Based on their biological characteristics, the State^(16)^ intervenes in populations, welcoming them under different policies, in a general political strategy of power. The life of each person becomes an object of control, with population variables such as birth rate, life expectancy, among others.

To regulate, control, record, and intervene in population variables, biopolitical agents are necessary, who, within a health policy, are healthcare professionals. Their responsibilities in guiding the population may vary according to the new directions taken by health policy. In line with the findings of this research, when analyzing PNAB challenges, the following are highlighted: a lack of resources in a BHU to meet the community’s demand, staff turnover affecting continuity of care, devaluation of continuing education, and funding cuts, which directly impact resources to meet community demand and the health unit’s infrastructure^(17)^.

Nurses’ actions are central to the computerization process of SUS, as this allows for accumulation and recording of applied knowledge, as well as quantification of their practical work, which is essential to guarantee continued patient care. It is observed that record-keeping allows professionals to know users in greater detail through more complete information about their health situations^(18)^. However, the nurses in this article report feeling a sense of professional distance from users due to concerns about record-keeping, which aligns with other findings that highlight factors negatively impacting the use of e-SUS, such as inappropriate infrastructure and excessive demand in PC^(19)^.

Accumulation of knowledge, based on records, is problematized by Foucault^(16)^, when looking at the conduct of the late 16^th^ and early 17^th^ centuries in France and England, where birth and mortality statistics were established. In that period, the State’s sanitary concern was expressed through these numbers to consider the population’s health index and population growth without, however, developing any effective or organized intervention to raise its health level. This accumulation of knowledge^(20)^ belongs to the health planning process. The analysis of this compilation of population information and the capacity to offer services in the territory translates, through quantifiable means, what the health needs of the State are.

The recording of this data serves to establish goals, produce new standards and protocols, and foster indicators that demonstrate and prove what is being done in their practices, as well as to classify and monitor the individuals assisted, thus generating funding for policies. The feeling reflected in nurses’ statements is that there is control over their practice in pursuit of numbers that are difficult to achieve.

Research analyzing the discourses of municipal managers in Piauí regarding the PC funding program in effect during that period demonstrated that, despite the motivation for financial return from productions, there is an establishment of control relationships, with regulations and penalties if agreements with the institution are not respected^(21)^. This resembles panopticism, which allows for the enhancement of a set of power relations, medical assistance, increased production, ease of instruction, and reduction of public spending, through an architectural structure that allows the constant passage of any inspector or academic to record the results of their experiment. It can be implicated in regulatory and supervisory roles^(22)^.

When comparing conventional BHU to FHS, nurses discussed the possible surveillance through CHWs. They pointed out the control of bodies through health practices such as measuring data and profiling. Healthcare professionals working in a traditional model in the municipality of Uberlândia also questioned the fact that it is not possible to classify their territory or know the individuals they serve, relating this to the lack of CHWs^(23)^. This direct surveillance permitted by FHS model is configured as a biopolitical strategy. It relates to a silent and discreet game that allows observing, monitoring, and controlling individuals^(22)^. Its hierarchical perspective establishes a unidirectional control, exercised by managers, representatives of the organization, over those who allow themselves to be controlled and submit to health work.

When they mention and compare tools existing in conventional BHUs and FHSs, they demonstrate the exercise of biopower. This tool has been used since the birth of modern states, being constituted from the emergence of the population, aiming to manage its security and its life through control techniques, disciplinary techniques, normalization and shaping of people’s behavior^(16)^.

In their conventional model, professionals feel authorized in their practices by current policies and the way their actions should occur. They speak of “open doors” because they receive everyone and do not know their territory, but also about an “active search” for certain users. When considering knowledge of the territory, study^(24)^ discussed ways of operating based on existing health model formats and their transitions over the years. FHS model prioritizes health promotion, prevention, and rehabilitation actions, focusing on promotion in the teams’ work process. The territorial linkage of the teams and the assignment of CHWs to a specific number of users allows actions to be organized according to community needs, with authorization to enter homes, collect information from individuals, and constantly monitor them.

The shortage of healthcare teams is one of the obstacles to carrying out planned activities, such as conducting group sessions. The inability to meet demands and the lack of strategies beyond the care that arrives at the units cause professionals to feel illegitimate in conducting their practice, as it does not align with the established model and they are not “authorized” to perform certain actions aimed at physicians.

State intervention in the pursuit of building social order occurs primarily not through repressive apparatuses, beyond a biomedical model, but rather through subtle control mechanisms operated by specialists, such as professionals from FHS teams, Expanded Family Health Centers, and PC teams, who aim to modify individuals’ and families’ behaviors, promoting the adoption of healthy habits. These practices are now seen as devices of social medicalization, as they emerge as strategies for controlling bodies in the fight against behaviors considered undesirable, controlling and medicalizing people’s conduct and attitudes^(25)^.

The realignments brought about by milestones in the history of PC policies propose other forms of care and generate discourses that produce insecurity among professionals, a lack of direction, and an inability to meet demands, highlighting losses and threats to the future when compared to how things were done before, perhaps thinking back to the first policy drafted.

Based on the premise of citizen well-being, various programs and policies are conceived in pursuit of social order, offering healthcare services that can be consumed. Built by the State upon the citizens, as an indispensable element of capitalism, at the cost of inserting bodies into the apparatus of production, health actions have become allied with the State, which has become a major provider, influencing and being influenced in this relationship in which it sometimes competes with, and sometimes assists, the public power in achieving the population’s health objectives^(15)^.

Nurses are subjectified by policies and programs, influenced by discourses and their reformulations in practice. They apply knowledge according to guidelines, focusing on their pronouncements to address different individuals’ potential needs, enabling them to achieve physical health and impacting the goals set out in health policies. One of the tools they use is group settings, a space where they provide guidance, prescribe care, and implement practices such as complementary therapies. Healthcare professionals trained in integrative practices demonstrate that these therapies can be applied collectively or individually, as needed^(26)^.

The governance of populations and practices of controlling individual behavior allow us to understand new dynamics, which are no longer based on imposing lifestyles, but rather move into the realm of self-governance^(14)^. This axis discusses how FHS intervenes in users’ lives through bodily practices. For instance, beyond biological parameters, professionals report that such practices are disciplinary devices over the body as a political and social being, thus controlling attitudes and behaviors, aiming for economically profitable and standardized bodies^(25)^.

The possibility of increased surveillance of individuals is perceived by nurses through CHWs, who enter the homes of those being assisted, observing the house conditions, organization, and bringing the team along. CHWs evoke popular knowledge and technical health expertise, occupying a strategic position, acting as an intermediary between the community and the healthcare service^(27)^.

The many functions mentioned are supported by PC health policies, in which nurses are placed at the center of processes, coordinating other professionals in their conduct, intervening in the relationship between the institution and professionals, and ensuring that what is published by health funding agencies is fulfilled. Many of nurses’ responsibilities, especially the general ones, could be performed by other team members so that clinical practice in relation to care does not take a back seat^(9)^.

The relationship between space and structure for the exercise of nursing is established not only between the nurse and the individual to whom care is directed, but also between the nurse and the object of care. This space is occupied by individuals authorized to speak and provide care based on their knowledge, to organize the necessary tools, and to establish power relations, according to the discourses that permeate it. Several aspects are related to nursing practice. Therefore, it is necessary to invest in reducing precarious employment contracts, respecting labor rights and retaining professionals, with the aim of strengthening their connection with the community, a fundamental aspect for the fulfillment of FHS role^(28)^.

### Study limitations

This study is limited by its portrayal of only one municipality in southern Brazil. Although there are several reports, differences between municipalities are possible.

### Contributions to nursing, health, or public policy

For the nursing field, this work, through problematization, can lead to a process of reflection, allowing nurses to see themselves reflected in their own discourses and those of others, and to explore other possibilities within their actions. For academia, this work provides an opportunity to reflect on PC nurses’ practices while also encouraging new research.

## FINAL CONSIDERATIONS

This study analyzed PC nurses’ knowledge and practices in a municipality in southern Brazil. It was found that health policies determine how their knowledge and practices are operationalized, changing through restructuring and the challenges they face. Health actions concerning the bodies of women and children, instigated for centuries, still prevail, appearing in a consolidated form in nurses’ discourse.

As for practices, PC nurses’ multifunctionality was highlighted, moving them away from clinical practice in favor of bureaucratization, which, although computerized, still presents challenges in its execution, resulting in duplicated work. The constant demand for performance imposes an overload on these professionals and feelings of inadequacy in the face of the difficulty of achieving unattainable goals. Decentralizing common tasks to other team members can minimize overload.

## Data Availability

The research data are available in a repository: https://doi.org/10.48331/SCIELODATA.YZGZ7V.
