# Supplementary material for: Knowledge and practices that constitute nurses in Primary Care
Source: Rev Bras Enferm. 2026 Jul 31;79:e20250083. doi: 10.1590/0034-7167-2025-0083 (PMC13427020; doi:10.1590/0034-7167-2025-0083)
Supplement: Translation

# Translation: Saberes e práticas que constituem enfermeiras e enfermeiros da Atenção Básica

**DOI:** 10.1590/0034-7167-2025-0083pt

**Authors:** José Ricardo Guimarães dos Santos, Stefanie Griebeler Oliveira, Teila Ceolin, Fernanda Eisenhardt de Mello, Sidneia Tessmer Casarin, Franciele Roberta Cordeiro, Vania Dias Cruz, Adrize Rutz Porto

**Affiliations:** I Prefeitura de Alvorada. Alvorada, Rio Grande do Sul, Brasil; II Universidade Federal de Pelotas. Pelotas, Rio Grande do Sul, Brasil; III Universidade Federal do Pampa. Uruguaiana, Rio Grande do Sul, Brasil

**Keywords:** Enfermeiras e Enfermeiros, Atenção Primária à, Saúde, Enfermagem de Atenção Primária, Enfermagem Prática, Política de Saúde.

## Abstract

**Objetivos:**

analisar saberes e práticas que constituem os enfermeiros da Atenção Básica de um município do sul do Brasil.

**Métodos:**

pesquisa qualitativa de inspiração foucaultiana, com 31 enfermeiros de Unidades Básicas de Saúde, no período de janeiro e junho de 2022. Os dados foram gerenciados no *software* ATLAS.ti^®^ e submetidos à análise de conteúdo e problematização com ferramentas foucaultianas, como saber, poder, discurso e subjetivação.

**Resultados:**

os saberes e práticas dos enfermeiros se constituem a partir das políticas públicas, sobretudo aquelas direcionadas aos corpos das mulheres e das crianças. Incluem-se na organização e funcionamento das ações programáticas, direcionando-as para orientação de rede.

**Considerações Finais:**

os enfermeiros atuam como agentes biopolíticos, orientando as ações do Estado para a gestão da vida da população. A sobrecarga é resultado da cobrança pelos indicadores de saúde, multifuncionalidade e informatização da saúde. Descentralizar atribuições comuns a outros membros da equipe pode minimizar a sobrecarga.

## INTRODUÇÃO

Ao longo da história, a enfermagem teve diferentes configurações, adquirindo um caráter científico a nível internacional na modernidade, no século XIX, com Florence Nightingale, e no Brasil, com a criação da Escola de Enfermeiras do Departamento Nacional de Saúde Pública em 1922, atualmente conhecida como Escola Anna Nery^(1)^. Este caminho é marcado por visão biologicista, hospitalocêntrica e reducionista do saber, de forma organizada e disciplinada, perpassando períodos de guerra, mudanças no cenário político até a Reforma Sanitária brasileira, que (re)formulou as políticas brasileiras, modificando a atuação dos enfermeiros^(2)^.

Outro marco para a história da enfermagem brasileira foi a primeira edição da Política Nacional de Atenção Básica (PNAB), que apresentou os princípios gerais da Atenção Básica (AB) no Sistema Único de Saúde (SUS), reconhecendo os enfermeiros como indispensáveis nas equipes de Saúde da Família. As atribuições a eles determinadas contemplavam desde atendimentos individuais, coletivos e gerenciais até a participação no controle social^(3)^.

Na atualidade, após duas edições da PNAB, reconhece-se a legitimidade dos enfermeiros em desempenhar atividades de natureza educativa, assistencial e administrativa, uma vez que são responsáveis pela realização de visitas domiciliares, consultas de enfermagem, diagnóstico de território, acolhimento com escuta qualificada e classificação de risco, de acordo com protocolos estabelecidos, assim como planejamento, gerenciamento e avaliação das ações desenvolvidas por técnicos/auxiliares de enfermagem e agentes comunitários de saúde (ACSs)^(4)^.

Amparados pelas políticas públicas, os enfermeiros passam a intervir sobre domínios biológicos, respaldados por saberes científicos e técnicos que se estabelecem como um saber poder, criando mecanismos de controle e de dominação dos sujeitos, desde os serviços de saúde até o domicílio^(5)^. Desse modo, estudar os saberes e práticas que constituem os enfermeiros da AB permite que sejam observadas as políticas que estão mais ou menos consolidadas, com mais engajamento e envolvimento dos profissionais nas suas diretrizes e ações. Permitem também olhar para a forma de organização, funcionamento e deslocamento de programas, políticas ou redes.

Considerando que os enfermeiros exercem a profissão seguindo as diretrizes do SUS, é interessante escutar e analisar essas trajetórias. Esta problematização tende a desnaturalizar e identificar os deslocamentos que políticas e programas têm ao longo do tempo. Acredita-se que políticas que atravessam determinados corpos há mais tempo tendem a estar mais cristalizadas nas práticas e saberes que constituem os profissionais. Por isso, a intenção deste estudo não é a construção de uma história que mostra uma linearidade na atuação dos enfermeiros na AB, mas apontar as rupturas, os deslocamentos, os movimentos para formação de certos saberes e as práticas exercidas por eles nesse espaço. Diante disso, delimitou-se como questão de pesquisa: quais saberes e práticas constituem os enfermeiros da AB de um município do sul do Brasil?

## OBJETIVOS

Analisar os saberes e práticas que constituem os enfermeiros da AB de um município do sul do Brasil.

## MÉTODOS

### Aspectos éticos

Os dados apresentados são parte de pesquisa maior, a qual versa sobre a história dos enfermeiros de um município do sul do Brasil, que foi aprovada por um Comitê de Ética em Pesquisa, sob Parecer nº 5.173.063. Foram respeitados os preceitos éticos da Resolução nº 466/2012^(7)^. Os participantes foram identificados por código, com a letra E (de enfermeiro), seguido pela ordem numérica das entrevistas.

### Tipo de estudo

Trata-se de pesquisa qualitativa sob perspectiva problematizadora de inspiração foucaultiana. A problematização é caracterizada pela elaboração de domínio de fatos, práticas e pensamentos, que põem suspensas ações políticas de forma crítica e considerando o período histórico^(6)^. Foram seguidos os critérios do *checklist COnsolidated criteria for REporting Qualitative research*.

### Cenário do estudo

O cenário da pesquisa foi constituído pelas Unidades Básicas de Saúde (UBSs) de um município do sul do Brasil, e o período da produção dos dados foi entre janeiro e junho de 2022. Há 51 UBSs, sendo 38 na área urbana e 13 na área rural. Entre essas, 38 possuem Estratégia Saúde da Família (ESF), e 11 são modelo tradicional, uma com ESF/mista - UBS, que também realiza atendimento noturno à população - e uma com Estratégia de Agentes Comunitários de Saúde (EACS). Esta pesquisa contemplou 49 dessas UBSs, pois não incluiu os modelos ESF/mista e EACS.

### Fonte de dados

O macroprojeto teve como proposta abordar um enfermeiro de cada UBS, seja convencional ou ESF. Os critérios de inclusão foram atuar no mínimo um ano em ESF ou UBS convencional e ser o enfermeiro da UBS com maior tempo de atuação na AB. Nas UBSs com mais de um enfermeiro com os critérios previstos, foi considerado aquele que apresentava mais tempo de trabalho na AB no município. O critério de exclusão foi estar em licença ou férias.

### Coleta e organização dos dados

O convite para participação da pesquisa foi por ligação telefônica, mensagens pelo aplicativo *WhastApp* ^®^ ou contato presencial nas UBSs. Para o final do período de coleta de dados, as UBSs não respondentes de contatos anteriores receberam ainda mais 21 tentativas de contato, sendo 13 contatos realizados via mensagem instantânea por aplicativo *WhastApp* ^®^, quatro por ligação por telefone celular e quatro contatos presencialmente. Dessas unidades contatadas, apenas cinco aceitaram participar e seis não responderam, o que foi considerado uma recusa. Dessa forma, 31 enfermeiros foram participantes, sendo que 18 não demonstraram interesse em participar.

O trabalho de campo foi realizado por oito estudantes de enfermagem, os quais receberam capacitação de quatro horas para se inserir nesta etapa. A entrevista semiestruturada foi utilizada como técnica de produção de dados, realizada de forma presencial com gravador de voz, ou *online*, pela plataforma do *Google Meet* ^®^, usando o celular como gravador de voz. A entrevista foi composta por quatro eixos: eixo 1 - caracterização dos participantes; eixo 2 - contextualização das políticas públicas de saúde; eixo 3 - exercício do trabalho; e eixo 4 - espaço de atuação. O tempo médio entre as entrevistas foi de 32 minutos, e para maior fidedignidade das falas, elas foram transcritas na íntegra no programa *Microsoft Word* ^®^, totalizando cerca de 13 horas de entrevistas e 202 páginas de conteúdo. Para compor o material empírico utilizado nesta pesquisa, a partir das entrevistas realizadas, foram extraídas informações referentes aos eixos um ao quatro.

### Análise dos dados

Todas as informações produzidas foram codificadas pelo *software* ATLAS.ti^®^, versão nuvem. Os códigos foram temas que emergiram a partir dos passos da análise de conteúdo^(8)^, desenvolvida em torno de três polos cronológicos: pré-análise - leitura flutuante a partir do contato com os trechos dos das entrevistas, com suas primeiras impressões relacionadas aos seus objetivos, que auxiliou na codificação em *software*; exploração do material - leitura, aprofundamento e organização da análise, confirmando as categorias e subcategorias apontadas; tratamento dos dados e interpretação - transformação dos dados brutos em dados significativos, permitindo construção de estruturas que condensam e põem em relevo essas informações. Foram gerados quatro códigos, nomeadamente historicidades, prática, rupturas e saber, os quais foram problematizados com as teorizações foucaultianas de saber, poder, discurso e subjetivação. Após essas etapas, foram construídas as categorias.

## RESULTADOS

Nesta pesquisa, foram entrevistados 31 participantes, os quais têm sua caracterização apresentada no Quadro 1.

**Quadro 1:** Caracterização sociodemográfica das enfermeiras e enfermeiro do estudo, Pelotas, Rio Grande do Sul, Brasil, 2022

Código	Tempo de formação (anos)	Idade	Sexo biológico	Cor autodeclarada	Modelo da unidade de saúde	Carga horária
E1	14	40	Mulher	Branca	ESF	40 horas
E2	8	33	Mulher	Preta	Tradicional rural	30 horas
E3	7	36	Mulher	Branca	ESF	40 horas
E4	15	39	Mulher	Branca	ESF	30 horas
E5	8	30	Mulher	Preta	ESF	40 horas
E6	15	47	Mulher	Branca	ESF	40 horas
E7	9	31	Mulher	Branca	ESF	40 horas
E8	22	45	Mulher	Branca	Tradicional	30 horas
E9	15	35	Mulher	Branca	ESF	40 horas
E10	8	30	Mulher	Branca	ESF rural	40 horas
E11	15	38	Mulher	Branca	ESF	40 horas
E12	20	42	Mulher	Branca	Tradicional	40 horas
E13	10	52	Mulher	Branca	ESF rural	40 horas
E14	22	51	Mulher	Branca	ESF	40 horas
E15	6	30	Mulher	Branca	ESF	40 horas
E16	12	38	Mulher	Branca	ESF	40 horas
E17	16	40	Mulher	Parda	ESF	40 horas
E18	26	50	Mulher	Branca	Tradicional	36 horas
E19	18	44	Mulher	Branca	ESF	40 horas
E20	20	41	Mulher	Branca	ESF	40 horas
E21	4	28	Mulher	Branca	ESF	40 horas
E22	15	38	Mulher	Branca	ESF	40 horas
E23	21	43	Mulher	Branca	ESF	40 horas
E24	12	48	Homem	Branca	ESF rural	40 horas
E25	8	34	Mulher	Branca	ESF	40 horas
E26	8	37	Mulher	Branca	ESF	40 horas
E27	28	56	Mulher	Branca	ESF	40 horas
E28	11	47	Mulher	Parda	Tradicional	30 horas
E29	36	57	Mulher	Branca	Tradicional	40 horas
E30	12	39	Mulher	Branca	ESF	30 horas
E31	35	57	Mulher	Branca	ESF rural	40 horas

*ESF - Estratégia Saúde da Família; E - enfermeiro.*

Entre os participantes, 30 (96,7%) são mulheres, com idades entre 28 e 57 anos, com cor autodeclarada, em sua maioria (87%), branca. Além disso, 87% dos enfermeiros possuem vínculo por regime estatutário. Sobre a carga horária, a maior parte (80,6%) trabalha 40 horas semanais. Em relação ao modelo da UBS em que trabalham, 80,6% atuam em ESF, e 19,3%, em UBS tradicionais, sendo 12,9% ESF rural, e 3,2%, tradicional rural.

A seguir, serão apresentadas as seguintes categorias elaboradas: Historicidades: políticas de saúde da Atenção Básica - “das gavetinhas às redes”; Registros, informatização e burocratização - “o e-SUS funciona quando quer”; Unidade Básica de Saúde convencional “porta aberta” e as (im)possibilidades na Estratégia Saúde da Família; e Multifuncionalidade do enfermeiro na Estratégia Saúde da Família *versus* a “infraestrutura mínima”.

### Historicidades: políticas de saúde da Atenção Básica - “das gavetinhas às redes”

Ao pensarem na (des)construção das políticas de saúde vigentes durante seu tempo de atuação e seus impactos ao longo da sua trajetória, as enfermeiras e o enfermeiro trouxeram suas percepções em relação às modificações em suas atribuições e suas práticas. Inicialmente, com ações ou programas destinados a esta ou aquela população, com a formulação das políticas, observa-se que elas têm efeito de direcionar e de conduzir os enfermeiros para organizar e estruturar a oferta de cuidados para determinadas populações.

*Logo no início, até pouco tempo atrás ainda, parecia umas gavetinhas, saúde da criança, saúde da mulher. Saúde do homem quase não se fala, não se falava, se tratava como saúde do adulto. Doenças sexualmente transmissíveis, ou AIDS, agora é IST.* (E12)

*Inicialmente, era esse programa que existia: a puericultura que era o cuidado com as crianças pequenas, com vacinação, acompanhamento do crescimento, desenvolvimento e o pré-natal das mulheres das gestantes. E os programas foram sendo acrescentados, com o decorrer do tempo, depois com a municipalização dos programas.* (E29)

*Melhorou muito quando começou a Rede Cegonha, nos ajudou muito sabe para estruturar o serviço em saúde da mulher e da criança. Minha formação foi bem diferente, quando eu fiz a graduação era totalmente diferente e aí com a Rede Cegonha a gente começou meio que pensar junto sabe, mãe e filho e olhar diferente.* (E23)

A transformação para um modelo em rede também ampliou as atribuições dos enfermeiros, para que pudessem, no campo da assistência, realizar consulta de enfermagem e visita domiciliar.

*Os enfermeiros trabalharem na assistência, hoje, a gente vê os enfermeiros fazendo consulta de enfermagem e visita domiciliar, e a gente acha que é tranquilo, mas isso, pra nós, foi uma conquista. Porque quando nós entramos na rede, nós recebemos cinco unidades para sermos responsáveis, para gente ficar um dia em cada unidade.* (E29)

*Aí esse governo, do PDT, ele expandiu para 52 unidades. Foi muito interessante, mas aquele formato eram UBS todas, não existia PSF. Aí trabalhava ali o clínico, o pediatra, às vezes um gineco, era o básico do básico. Tinha uma sala de vacinas que era usado ali tudo junto para a puericultura. E para a enfermagem, eu acho que tinha outra de procedimentos.* (E23)

### Registros, informatização e burocratização - “o e-SUS funciona quando quer”

A informatização representou sobrecarga no fazer profissional. Relatos mostram que parece haver problemas no armazenamento dos dados e, muitas vezes, os sistemas não apresentam um bom funcionamento, gerando a necessidade de registros duplicados em meio físico e eletrônico:

*A saúde da criança, por exemplo, a gente tem que saber organizar, vê se a gente tá com tudo em dia, vacinação. A gente não pode contar com o e-SUS, porque funciona quando quer. Eu não uso físico* [prontuário], *porque eu acho que tem que ser abolido.* (E14)

*O sistema E-SUS tu cadastra e cadastra, e de 100, são computados tipo 40 ou 50.* (E26)

*Tu perdes um pouco da excelência do trabalho, porque tu ficas só preocupada com preencher e olha para criança, para a mãe, para a mulher que veio fazer CP.* (E14)

A informatização é percebida como um meio de controle, pois, pelos dados registrados em prontuário eletrônico, os gestores extraem informações para cobrança dos enfermeiros e suas respectivas equipes, que percebem esses indicadores como impossíveis diante das suas realidades.

*Qual é o sentimento que eu tenho? Que as coisas estão se terminando. Que existe uma cobrança gigantesca por controle de dados, por alcançar metas, por provar atendimentos, e aí a gente um tipo é uma cobrança e não. Se tem respaldo, vamos supor, eu tô há cinco anos com uma microárea completamente sem ACS - a microárea mais vulnerável do território. Aí redistribuí os ACSs. Ano passado, uma das melhores ACS se aposentou; eu fiquei com duas áreas descobertas.* (E26)

*Isso já é oficial, serão 12 ou 13, mas não se sabe qual serão os outros indicadores. Tem indicadores que eles já criaram para o pessoal não conseguir metas. A meta é o seguinte: tu atingiste a meta, o município vai ganhar um X. Não atingiu? Não ganha.* (E24)

### Unidade Básica de Saúde convencional “porta aberta” e as (im)possibilidades na Estratégia Saúde da Família

Alguns participantes trabalham em UBS no município com o modelo tradicional e trazem, a partir de suas experiências, a descrição de suas práticas em comparação com as de uma ESF, bem como algumas de suas limitações, como não conhecer o perfil do usuário para o planejamento da oferta de cuidados, não realizar visitas domiciliares, não ter composição de grupo e não ter a equipe completa, devido à ausência do ACS no quadro funcional, o que centraliza as funções no enfermeiro.

*Na estratégia, a gente tem equipes parece mais completas* [...] *então tu consegues te articular melhor. O protocolo novo de acolhimento* [...] *ele foi feito para, por exemplo, o primeiro atendimento não ser feito só por enfermagem. Ele pode ser realizado por qualquer profissional de nível superior da unidade* [...] *e a gente não consegue fazer isso, sempre acaba ficando centrado no enfermeiro por N fatores* [...]. (E2)

*Eu acho mais interessante, tu crias vínculos. A gente cria vínculo, vê a criança crescer, vê o desenvolvimento, vê algumas coisas que a gente observou e passou para o médico e foi resolvido porque nós vimos.* (E8)

*O que diferencia? A população em si não é delimitada. Por exemplo, na estratégia, tu tem tantos habitantes. Tu sabes quantas gestantes tu tens, quantas crianças tu tem, como organizar de uma maneira que fique mais prática. Aqui, por exemplo, se tu me perguntar quantas gestantes tu tem, a gente tem em média 200 gestantes, mas em média.* (E18)

Os profissionais discutiram acerca da reorganização de suas rotinas na UBS convencional partindo da premissa que são um serviço de “porta aberta” e precisam absorver as demandas que chegam, com impasses para busca ativa e realização de visitas domiciliares.

*A gente não faz visita domiciliar. Em uma unidade tradicional, não teria essa necessidade, mas quando há uma situação que necessita, a gente faz porque tem um motorista.* (E2)

*Na UBS convencional onde eu estou agora, a gente não tem como fazer uma busca ativa, por mais que eu tenha vínculo. E como a gente é “porta aberta”, a gente recebe de todos os lugares as pessoas, e quando se trata de busca ativa, não tem muito como recorrer. A parte de visita domiciliar também é uma atividade que raramente se faz.* (E12)

Quando refletiram sobre as dificuldades em suas práticas na ESF, as enfermeiras e o enfermeiro apontaram como (im)possibilidade para ações como grupos, de maneira geral, o impacto da falta de profissionais de diferentes categorias:

*A gente tenta fazer dentro do possível os eixos prioritários da estratégia. A gente sente falta dos grupos. E a gente não consegue em função da equipe defasada.* (E1)

*Nesse momento, a gente anda sobrecarregado e também com um desfalque de colegas, de todos os profissionais. Não existe uma equipe que esteja completa na cidade.* (E23)

*Eu, por sorte, atualmente, tenho médico, mas, para teres ideia, várias equipes da zona rural, com equipes incompletas, não têm médico, ou seja, fica difícil tu trabalhar, fazer um Programa de Saúde da Família se a equipe está incompleta.* (E24)

As mudanças previstas nas políticas públicas, observadas ao longo dos anos de atuação, também foram apontadas pelas enfermeiras e pelo enfermeiro por gerar entraves em seu exercício, pois, para além das dificuldades de execução, eles seguem caminhos contrários aos propostos em sua construção, causando inseguranças por não permitirem que atuem conforme as cartilhas e manuais.

*A impressão que eu tenho é que o SUS tá acabando, que a ESF tá acabando. É esse o sentimento que eu tenho todos os dias quando eu venho trabalhar: de que a gente tá tentando segurar um barco que tá afundando. Um barco furado, e a gente de baldinho tentando tirar a água, só que entra mais água do que a gente consegue tirar.* (E23)

*Eu acho que as políticas de saúde, principalmente as políticas de saúde da família, são bem importantes, só que, nos últimos anos, vêm sofrendo grandes mudanças, inclusive a Estratégia Saúde da Família eu vejo que parece que está perdendo o “norte”. Tem coisas mudando muito, e eu fico preocupado com o futuro da saúde da família.* (E24)

Entretanto, algumas falas demonstram que, apesar dos atravessamentos na ESF, há possibilidades e potencialidades encontradas em suas práticas para que realizem alguns eixos, mesmo que diferente do que deveria ser e/ou está escrito nos cadernos da política de AB. Como exemplo, citaram os grupos, o Programa Saúde na Escola e visitas domiciliares.

*Aqui, a gente bota em prática todas as políticas públicas, não da maneira que deveria ser, mas sim, a gente tem a puericultura, que a gente coloca em prática a Caderneta de Saúde da Criança, o Caderno de Atenção Básica. Então, tudo que consta ali a gente tenta realizar. Na saúde da mulher, a mesma coisa. Nas coletas de citopatológico, nos pré-natais de baixo risco, a gente faz todo esse acompanhamento e utiliza os cadernos de atenção, que é o material que a gente tem pelo ministério.* (E5)

*Agora tá começando o grupo de gestantes de novo. A gente não consegue fazer muita coisa. Tem o PSE, que a gente faz saúde na escola. A gente tem seis escolas vinculadas à nossa unidade, então a gente dá uma atenção maior para essas crianças também. Visita domiciliar, a gente faz toda quinta a tarde. Se tiver paciente ou alguma demanda que venha da rua, a gente se organiza e sai às 11:00 para organizar as visitas.* (E7)

Além disso, os enfermeiros reconhecem a ESF como estratégia que promove maior vínculo com o usuário, que exige maiores responsabilidades, conhecimento de sua família, o contexto, situação de saúde e o ACS - o sujeito que intermedia essa relação.

*Na ESF, estamos muito mais conectados com a família. A responsabilidade é bem maior* [...] *os ACSs vão nos trazendo as demandas* [...]. (E4)

*A presença dos ACSs quando eles são atuantes, pois faz muita diferença pela criação do vínculo. Ele traz em reunião muitos temas que, por exemplo, a pessoa vem para o atendimento e tu conhece a pessoa naquele momento do atendimento, mas não conhece o contexto dela. Então, o ACS diz: “Olha, não tem como alguém prescrever a medicação tal para o paciente tal, porque a casa dele é um quadrado desse tamanho e ele mal tem o que comer e o paciente tem vergonha de dizer”. Tem gente que diz: “Olha, se esse remédio for caro, eu não tenho como comprar”, mas tem gente que não fala isso.* (E5)

### Multifuncionalidade do enfermeiro na Estratégia Saúde da Família *versus* a “infraestrutura mínima”

Os muitos papéis exercidos pelos enfermeiros na ESF também são postos a jogo em suas falas, ressaltando a centralização das atribuições administrativas a esses profissionais, que ficam responsáveis em gerenciar a UBS, seus insumos e a manutenção do local junto à sua prática assistencial.

*Porque fica tudo em cima do enfermeiro. Eles não distribuem as atribuições. Tudo que aparece de novo é o enfermeiro, que é o responsável por aquilo, desde pedido de troca de lâmpada até um pré-natal de baixo risco, e eles querem que a gente dê conta disso nessas oito horas de trabalho diária, e aí, às vezes, fica bem difícil. Tu perde uma manhã toda com acolhimento, e aí tem a época que tem que fazer o pedido, que tem que controlar os CPs* [exame citopatológico de colo uterino]. *É várias demandas administrativas que sobrecarregam demais.* (E3)

*Eu tenho uma penca de relatórios para fazer, fazer escala. Eu tenho que fazer o ponto; eu tenho que* [...] *um funcionário entrou de férias, de licença de não sei o que, a gente tem que ver a burocracia. Não sobra muito tempo para gente* [...] *a gente acaba atendendo mais a demanda, apagando incêndio do que vem. Tudo passa pelo enfermeiro: é a criança que chega com febre, que tá com bronquite, é feridinha, é tudo.* (E9)

*É que o enfermeiro, na rotina de uma unidade, ele gerencia tudo. Desde pedido de medicação, pedido de insumos, conserto, manutenção, os controles, quem são as gestantes. Se elas estão vindo ou se elas não tão. Quem são as crianças de puericultura. Ter esse respaldo de que o ACS tá dando conta da sua área e está conseguindo quebrar o galho daquela outra que não tem ninguém.* (E26)

Além das muitas funções exercidas, a relação com espaço físico e a impossibilidade em ofertar cuidados previstos pelas políticas e protocolos vigentes, como o acolhimento e a organização do espaço que possa circular o ar e otimizar o atendimento a todos os usuários que chegarem no local, são destacados nas seguintes falas:

[...] *acredito que, pelo tanto de profissional que trabalha aqui, poderíamos ter uma unidade maior. Temos duas salas de enfermagem: uma de vacina e outra para consulta de enfermagem. Somos quatro enfermeiros por turno, então não temos espaço para todos atuarem. A gente mesmo acaba sentando na farmácia* [...] *tínhamos que ter uma ampliação pensando na equipe. Para o usuário, diminuiria o tempo de espera* [...]. (E4)

[...] *infraestrutura mínima. Tem profissionais para atender; não temos espaço físico; não conseguimos atender às pessoas de acordo com o protocolo necessário, por exemplo, atender às pessoas numa sala com a porta fechada. Ventilação, tem salas que a gente adaptou que não tem janela nem porta para poder pelo menos fazer uma escuta de um paciente e otimizar o nosso tempo* [...]. (E11)

[...] *não tem sala de enfermagem* [...] *agora mesmo, eles querem implementar o acolhimento, mas não têm sala para o acolhimento. Enfermeiro não tem sala, e isso é uma coisa que é banalizada há anos* [...]. (E14)

## DISCUSSÃO

Observa-se predominância de mulheres entre os participantes, o que tende a direcioná-las para citar ações programáticas para mulheres e crianças. A média de tempo de formação foi de 12 anos, o que permite identificar mudanças de organização e funcionamento nas ações programáticas com direcionamento para orientação de rede. Isso amplia o olhar dos enfermeiros sobre as pessoas adscritas em seu território de atuação. Entre os desafios, destacam-se a sobrecarga profissional mediante a informatização e burocratização, e a multifuncionalidade do enfermeiro.

Acerca das movimentações na organização do serviço de saúde, a menção de “gavetinhas”, que comportavam o homem, a mulher e a criança, mostra o retrato de uma saúde fragmentada. De programas específicos para redes de atenção à saúde, nota-se que as práticas discursivas e não discursivas se interligam para aumentar os níveis de saúde da população. Em relação a isso, enfermeiros da AB, atuantes na saúde da família ou na convencional, demonstram que as ações mais desempenhadas incluíram a puericultura e a prevenção do câncer de colo de útero^(9)^.

A Rede Cegonha foi citada por enfermeiros como exemplo de política que permitiu estruturar cuidado e vigilância sobre o corpo da mulher, surgindo pautada por eixos^(10)^ de humanização e integralidade na saúde. Em seus dez anos de implementação^(11)^, é tida como efetiva, pois incluiu a presença do companheiro ou outro familiar de escolha das mulheres, consistindo em estrutura^(12)^ composta por locais e agentes voltados para atender à gestação, ao parto e ao puerpério de baixo risco. Para Foucault^(13)^, os primeiros corpos que receberam intervenções estratégicas objetivando sua produtividade foram mulheres saudáveis e nascimentos de bebês com a mesma vitalidade.

A biopolítica visa à espécie humana e à população, e é uma tecnologia de poder que objetiva regular a vida cotidiana. Essa forma de poder é utilizada por instituições diversas, operando por meio da segregação, organização dos corpos e da hierarquização social, garantindo as relações de dominação^(14)^. Nesse sentido, a organização das políticas de saúde em redes viabiliza o exercício da biopolítica junto aos profissionais de saúde, que conduzem os níveis de saúde da população, mesmo com traços persistentes do sistema fragmentado.

Os enfermeiros evocaram os modelos de saúde e suas transições, exemplificando que o Programa de Saúde da Família se estruturou com objetivos traçados, tornando-se, mais tarde, uma estratégia que abarcou novas maneiras de (re)produzir saúde, conhecer o território e o perfil dos sujeitos. No entanto, o conjunto de demandas exigidas ao enfermeiro não é plenamente executado, uma vez que a ele cabe integrar a equipe de saúde e também desenvolver o seu fazer profissional^(9)^.

As configurações nessas políticas têm um direcionamento, que é a população. Para Foucault^(15)^, a população não consiste em indivíduo-corpo, mas sim um corpo com inúmeras cabeças, se não infinito, pelo menos necessariamente numerável. Para conhecer esses corpos, há o exercício do poder, que permeia, produz coisas, forma saber e produz discurso. Partindo de suas características biológicas, o Estado^(16)^ intervém sobre populações, acolhendo-as sob diferentes políticas, em uma estratégia política geral de poder. A vida de cada pessoa passa a ser objeto de controle, com as variáveis da população, tais como aspecto de natalidade, expectativa de vida, entre outros.

Para regular, controlar, registrar e intervir nas variáveis da população, são necessários os agentes biopolíticos, os quais, dentro de uma política de saúde, são os profissionais de saúde. Suas atribuições para conduzir a população podem variar conforme os novos direcionamentos que são tomados pela política de saúde. Convergindo com os achados desta pesquisa, ao analisar os desafios da PNAB, apontam-se a falta de recursos em uma UBS para atender à demanda da comunidade, rotatividade dos profissionais, que afeta a longitudinalidade, desvalorização da educação permanente e corte no financiamento, que implica diretamente nos recursos para atender à demanda da comunidade e na infraestrutura da unidade de saúde^(17)^.

As ações dos enfermeiros estão incumbidas no processo de informatização do SUS, pois isso permite o acúmulo e o registro dos saberes aplicados, além da quantificação do que produzem em suas práticas, o que é essencial para garantir o cuidado continuado aos pacientes. É visto que o registro permite ao profissional conhecer mais detalhadamente o usuário por meio de informações mais completas sobre as situações de saúde^(18)^. Porém, os enfermeiros do presente artigo relatam sentimento de distanciamento profissional dos usuários pela preocupação com os registros, o que vai ao encontro com outros achados que apresentam fatores que interferem negativamente na utilização do e-SUS, como infraestrutura inapropriada e excesso de demanda na AB^(19)^.

O acúmulo de saberes, a partir dos registros, é problematizado por Foucault^(16)^, ao olhar para as condutas do final do século XVI e começo do século XVII na França e Inglaterra, em que se estabeleceram estatísticas de nascimento e mortalidade. Naquele período, a preocupação sanitária do Estado se dava através desses números para pensar o índice da saúde da população e do aumento da população sem, entretanto, desenvolver alguma intervenção efetiva ou organizada para elevar o seu nível de saúde. Esse acúmulo de saber^(20)^ pertence ao processo de planejamento de saúde. A análise desse compilado de informações da população e a capacidade de oferta de serviços no território traduzem, pelo quantificável, quais as necessidades de saúde do Estado.

O registro desses dados serve para estabelecer metas, produzir novos padrões e protocolos e fomentar indicadores que demonstrem e comprovem o que está sendo feito em suas práticas, bem como para classificar e realizar o acompanhamento dos indivíduos assistidos, gerando assim financiamento para as políticas. O sentimento refletido nas falas das enfermeiras e do enfermeiro é de que há um controle sobre seu exercício em busca de números difíceis de alcançar.

Pesquisa que analisou os discursos de gestores municipais do Piauí sobre o programa de financiamento da AB vigente no período demonstrou que, apesar da motivação pelo retorno financeiro por produções, existe o estabelecimento de relações de controle, com normativas e punições caso não sejam respeitadas as pactuações com a instituição^(21)^. Isso se assemelha a um panoptismo, que permite o aprimoramento de um conjunto de relações de poder, assistência médica, aumento da produção, facilidade de instrução e redução dos gastos públicos, por meio de uma estrutura arquitetônica que permite a passagem constante de qualquer inspetor ou acadêmico para registrar os resultados de sua experiência. Podem ser implicadas em normativas e fiscalizadoras^(22)^.

Ao aproximarem a UBS convencional à ESF, os enfermeiros discutiram sobre a vigilância possível através dos ACSs. Apontaram sobre o controle dos corpos por meio de práticas de saúde como mensurar dados e perfil. Profissionais de saúde que trabalham em um modelo tradicional do município de Uberlândia também problematizaram o fato de não ser possível classificar seu território, conhecer os sujeitos atendidos, relacionando isso à falta de ACS^(23)^. Essa vigilância direta permitida pelo modelo de ESF se configura como estratégia biopolítica. Ela se relaciona a um jogo silencioso e discreto que permite observar, fiscalizar e controlar os indivíduos^(22)^. Sua perspectiva hierárquica estabelece um controle unidirecional, exercido dos gestores, representantes da organização, sobre aqueles que se deixam controlar e se submetem ao trabalho em saúde.

Quando mencionam e comparam as ferramentas existentes nas UBSs convencionais e nas ESFs, demonstram o exercício de biopoder. Essa ferramenta foi utilizada desde o nascimento dos Estados modernos, sendo constituída a partir do surgimento da população, objetivando gerir sua segurança e sua vida por meio de técnicas de controle, técnicas disciplinares, normalização e moldagem das condutas das pessoas^(16)^.

Em seu modelo convencional, os profissionais se sentem autorizados em suas práticas pelas políticas vigentes e pela forma como suas ações devem ocorrer. Falam de “portas abertas” por receberem a todos e não conhecerem seu território, mas também sobre uma “busca ativa” de determinados usuários. Ao pensar no conhecimento do território, estudo^(24)^ discutiu sobre as formas de operar a partir dos formatos existentes dos modelos de saúde e suas transições ao percorrer dos anos. O modelo de atenção de ESF prioriza ações de promoção, prevenção e reabilitação em saúde, enfocando a promoção no processo de trabalho das equipes. A vinculação territorial das equipes e a vinculação dos ACSs a um número específico de usuários possibilitam que as ações sejam organizadas de acordo com as necessidades da comunidade, com a autorização de adentrar os domicílios coletando as informações dos indivíduos, os quais monitoram constantemente.

A defasagem nas equipes de saúde é um dos impedimentos diante das atividades previstas, como a realização de grupos. A impossibilidade de atender às demandas e a falta de outras estratégias além do atendimento que chega às unidades fazem com que o profissional não se sinta legitimado a conduzir sua prática, por não estar de acordo com o modelo previsto e por não estar “autorizado” a realizar algumas ações voltadas ao sujeito médico.

A intervenção do Estado, na busca de construção da ordem social, dá-se, sobretudo, não por aparelhos repressivos, para além de um modelo biomédico, mas sim por dispositivos de controle sutis, operados por especialistas, como profissionais das equipes de ESF, Núcleo Ampliado de Saúde da Família e AB, que objetivam modificar os comportamentos dos indivíduos e das famílias, favorecendo a inserção de hábitos saudáveis. Tais práticas passam a ser encaradas como dispositivos de medicalização social, pois surgem como estratégias de controle dos corpos no combate aos comportamentos considerados indesejados, controlando e medicalizando as condutas e atitudes das pessoas^(25)^.

Os realocamentos trazidos pelos marcos na historicidade das políticas da AB propõem outras formas de cuidado e geram discursos que produzem inseguranças nos profissionais, uma falta de “norte” e a incapacidade de darem conta das demandas, pontuando as perdas e as ameaças ao futuro, ao comparar ao como era feito, talvez pensando na primeira política redigida.

Na premissa do bem-estar do cidadão, vários programas e políticas são pensados, em busca de uma ordem social, com a oferta de uma saúde a ser consumida. Construídas pelo Estado sobre os cidadãos, como elemento indispensável do capitalismo, à custa da inserção dos corpos nos aparelhos de produção, as ações de saúde se aliaram ao Estado, que se tornou um grande provedor, influenciando e sendo influenciado nessa relação em que ora compete, ora auxilia o poder público na consecução dos objetivos de saúde da população^(15)^.

Os enfermeiros são subjetivados pelas políticas e programas, sendo atravessados pelos discursos e suas reformulações em prática. Aplicam os saberes conforme as cartilhas, debruçando-se sobre seus ditos para conduzir as possíveis necessidades dos diferentes sujeitos, sendo capazes de tornar seus corpos saudáveis, com impacto nas metas previstas nas políticas de saúde. Uma das ferramentas são os grupos, espaço em que fazem suas orientações, prescrições de cuidados e implementam práticas, como as terapias complementares. Profissionais de saúde formados em alguma prática integrativa demonstram que elas podem ser aplicadas de maneira coletiva ou individual, conforme a necessidade^(26)^.

O governo das populações e das práticas de controle das condutas dos sujeitos permite entender novas dinâmicas, as quais não estão mais pautadas em impor estilos de vida, mas passam para o campo do autogoverno^(14)^. É abordado como a ESF intervém na vida dos usuários através de práticas corporais. Por exemplo, para além de parâmetros biológicos, os profissionais relatam que tais práticas são dispositivos de disciplina sobre o corpo enquanto ser político e social, controlando, assim, suas atitudes e condutas, objetivando corpos economicamente rentáveis e normatizados^(25)^.

A possibilidade de uma maior vigilância sobre os sujeitos é percebida pelos enfermeiros através do ACS, que adentra o lar dos sujeitos assistidos, observando as condições da casa, sua organização e levando a equipe. O ACS evoca saber popular e saber técnico da saúde, ocupando posição estratégica, com papel de interlocutor entre comunidade e serviço de saúde^(27)^.

As muitas funções apontadas são sustentadas nas políticas de saúde da AB, em que o enfermeiro é colocado no centro dos processos, coordenando os demais profissionais nas suas condutas, intercedendo na relação entre a instituição e os profissionais, e assegurando que o que é publicado pelos órgãos de fomento à saúde seja cumprido. Muitas das atribuições do enfermeiro, considerando especialmente as gerais, poderiam ser desempenhadas por outros membros da equipe, para que a prática clínica em relação ao cuidado não fique em segundo plano^(9)^.

A relação entre o espaço e a estrutura para exercício da função de enfermeiro não se estabelece apenas entre este e o sujeito a quem os cuidados são direcionados, mas também entre este e o objeto. Este é ocupado por sujeitos autorizados a dizer e a prestar cuidados com base nos seus conhecimentos, a organizar as ferramentas necessárias e a estabelecer relações de poder, conforme os discursos que o atravessam. Diversos aspectos estão relacionados ao exercício do enfermeiro; por isso, é necessário investir na desprecarização dos contratos de serviço, respeitando os direitos trabalhistas e fixando os profissionais, com a condição para ampliar o vínculo com a comunidade, aspecto fundamental para a concretização do papel da ESF^(28)^.

### Limitações do estudo

Este estudo apresenta como limitação ter o retrato de apenas um município do sul do Brasil. Apesar de haver diversos relatos, é possível que haja diferenças entre municípios.

### Contribuições para a área da enfermagem, saúde ou políticas públicas

Para a área da enfermagem, este trabalho pode, a partir da problematização, ocasionar um processo de espelhamento, para que os enfermeiros possam se enxergar através dos discursos proferidos por si e pelos outros, e buscar fazer outros movimentos, exercendo outras possibilidades dentro de seus atos. Já para a academia, este trabalho oportuniza uma reflexão sobre as práticas dos enfermeiros na AB da mesma forma que instiga novas pesquisas.

## CONSIDERAÇÕES FINAIS

Este estudo permitiu analisar saberes e práticas que constituem os enfermeiros da AB de um município do sul do Brasil. Constatou-se que as políticas de saúde determinam a maneira pela qual os saberes e as práticas dos profissionais são operacionalizados, modificando-se mediante a reestruturação e os próprios desafios enfrentados. Ações de saúde sobre os corpos das mulheres e das crianças, incitadas há séculos, ainda prevalecem, apresentando-se de forma consolidada nas falas dos enfermeiros.

Em relação às práticas, destacou-se ainda a multifuncionalidade dos enfermeiros na AB, afastando-os da clínica em detrimento da burocratização, a qual, embora informatizada, ainda apresenta desafios em sua execução, repercutindo em trabalho duplicado. A permanente cobrança por desempenho impõe sobre esses profissionais uma sobrecarga e sentimentos de insuficiência diante da dificuldade de alcançar metas inatingíveis. Descentralizar atribuições comuns a outros membros da equipe pode minimizar a sobrecarga.

## Data Availability

Os dados de pesquisa estão disponíveis em repositório: https://doi.org/10.48331/SCIELODATA.YZGZ7V.
